# Effects of Temperature on Seed Germination of *Plantago lanceolata* and Management in *Carya illinoinensis* Production

**DOI:** 10.3390/plants8090308

**Published:** 2019-08-28

**Authors:** Timothy L. Grey, Kayla M. Eason, Lenny Wells, Nicholas T. Basinger

**Affiliations:** 1 College of Agriculture and Environmental Sciences, Department of Crop and Soil Sciences, University of Georgia, Tifton, GA 31793, USA; 2 College of Agriculture and Environmental Sciences, Horticulture Department, University of Georgia, Tifton, GA 31793, USA; 3 College of Agriculture and Environmental Sciences, Department of Crop and Soil Sciences, University of Georgia, Athens, GA 30602, USA

**Keywords:** (2,4-dichlorophenoxy)acetic acid, germination, halosulfuron-methyl, indaziflam, Lorentzian regression equation, *Plantago lanceolata*, simazine, thermal time

## Abstract

*Plantago lanceolata* L. (buckhorn plantain) is an encroaching winter weed described as one of the most successful noncultivated colonizing species around the world. Control of *P. lanceolata* in southeastern USA *Carya illinoinensis* (Wangenh.) K. Koch production has not been studied, nor has the role of temperature on germination using a thermal gradient table. Seed of *P. lanceolata* collected from a Georgia *C. illinoinensis* grove were tested for the effects of temperature over time to establish differences in effects on germination using a thermal gradient table. Temperatures ranged from 13.5 to 30.5 °C for 288 h. Cumulative *P. lanceolata* seed germination was 66% occurring at 17.8 °C at 242 h. Over the 288 h experiment, maximum *P. lanceolata* germination was 27% occurring at 17.0 °C, 187 h after initiation. Control of *P. lanceolata* with residual herbicides, or in combination with 2,4-dichlorophenoxyacetic acid (2,4-D) was evaluated in the interrow of *C. illinoinensis* groves containing *Trifolium repens* L., and in greenhouse experiments. Pre- and post-emergent herbicides included indaziflam, halosulfuron-methyl, and simazine applied alone, or in combination with 2,4-D in late autumn after *P. lanceolata* emergence in a *C. illinoinensis* grove. Indaziflam in combination with 2,4-D controlled *P. lanceolata* greater than 90% when applied in *C. illinoinensis* groves and greenhouse experiments. Halosulfuron-methyl and simazine applied alone, or in combination with 2,4-D, provided 67% or less *P. lanceolata* control in the grove experiments, and 83% or less in greenhouse experiments. Results suggested that herbicide applications should be made during the time when diurnal temperatures are between 15 and 30 °C, while abiding pre-harvest interval restrictions. Post- and pre-emergent herbicides may aid in controlling emerged weeds and reducing further weed emergence during the autumn of that year.

## 1. Introduction

In the southeastern USA *Carya illinoinensis* (Wangenh.) K. Koch production region of Alabama, Florida, Georgia, North Carolina, and South Carolina, winter weed species, such as *Plantago lanceolata* L. (buckhorn plantain), can be an issue from autumn to spring of the interrow vegetative strips [1]. Generally, autumn herbicides cannot be applied due to pre-harvest intervals as growers are in the process of mechanically gathering the crop from the groves soil surface from September to January. One option is to apply herbicides post-emergence with soil residual activity, soon after harvest is completed to prevent weed species from proliferating.

Additionally, *C. illinoinensis* growers want to promote *Trifolium* species growth in groves, as a natural N source, and as a pollinator-friendly species. As growers continue to have issues with weeds that encroach into groves, they need solutions to maintain profitability. However, due to the perennial nature of this system, conditions and management practices are often favorable to the establishment and proliferation of *P. lanceolata* throughout a *C. illinoinensis* grove.

**Figure 1:**
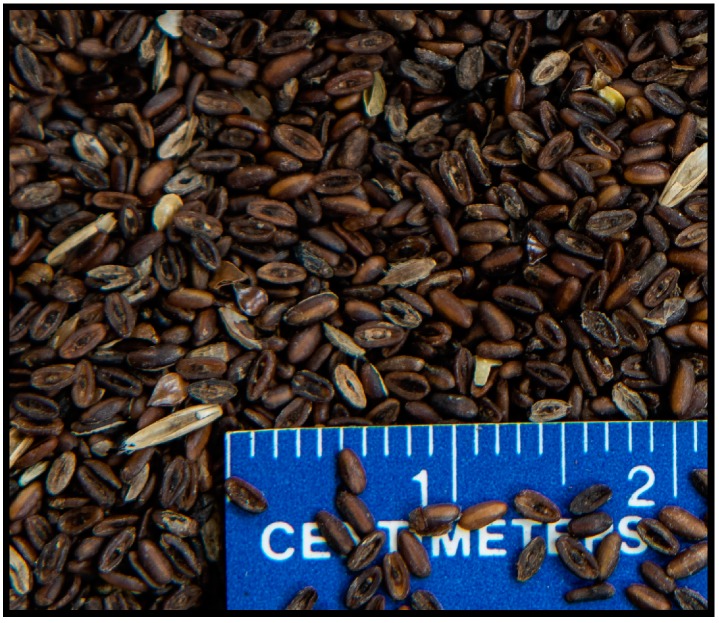
Seed utilized in germination testing on the thermogradient table and greenhouse experiments. Seed size of *Plantago lanceolata* L. from Webster County Georgia, USA (photograph by Sidney Cromer, used with permission).

*Plantago lanceolata* is an erect perennial [2] but often acts as an annual herb that can produce a greater number of seeds per unit leaf area when compared to other perennial weedy *Plantago* species [3]. It has become an issue in several *C. illinoinensis* growing areas as an encroaching winter weed species and has been a common and troublesome weed in tree fruit and nut surveys [4,5,6]. Described as one of the most successful noncultivated colonizing species [7], it begins to establish in late summer/autumn as days begin to shorten after the solstice, forms a vegetative rosette, followed by a seed stalk in the spring. *Plantago lanceolata* seed germination is not influenced by light as significantly as other *Plantago* species [8,9,10,11]. Research has indicated that temperatures between 20 to 25 °C produce the greatest germination in forages for this weed [11]. Thompson and Grime [12] noted that seed was insensitive to the presence or absence of light and temperature fluctuations that would simulate germination after storage at 20 °C. In the southeastern USA, *P. lanceolata* establishes in the autumn and continues to grow throughout the winter months, with increased foliar and taproot growth, and seed reproduction in the spring. Seed is spread as a mechanism of proliferation, similar to what occurs in turf settings [13]. It is well adapted to non-cultivated areas such as *C. illinoinensis* groves from roadside areas. Seeds are easily spread due to their small size (Figure 1) and carried on field equipment. 

Due to the timing of *P. lanceolata* emergence, management of this weed by using herbicides can be challenging. While 2,4-dichlorophenoxyacetic acid (2,4-D) is registered for post-emergence application in *C. illinoinensis*, there is growing concern over off-target movement, and 60-day pre-harvest intervals, which may not allow for the use of this product during *P. lanceolata* establishment [14]. Timing of pre- and post-emergent herbicide applications can be restricted by pre-harvest intervals and can limit the ability to control weeds. Registered pre-emergent herbicides that provide residual control in *C. illinoinensis* include indaziflam, simazine, and halosulfuron-methyl [15]. Combinations of 2,4-D with residual herbicides post-emergent have been shown to increase *P. lanceolata* control in turfs [13]. The inclusion of the residual herbicides indaziflam, simazine, or halosulfuron-methyl in conjunction with post-emergent applications of 2,4-D may provide the control needed to manage *P. lanceolata* while also complying with necessary pre-harvest interval requirements. 

The first objective for this research was to evaluate *P. lanceolata* seed morphology after mechanical collection, then determine the optimal time and temperature needed for that seed to germinate in laboratory experiments. If the time and temperature of *P. lanceolata* seed germination can be predicted, then using environmental data from regional weather stations may assist in qualifying its establishment under the canopy of *C. illinoinensis* trees. The second objective was to evaluate the efficacy of combinations of residual pre-emergent herbicides registered for use in *C. illinoinensis* with and without 2,4-D for control of *P. lanceolata* with autumn applications prior to nut harvest.

## 2. Results

### 2.1. Seed Morphology

Average *P. laceolata* seed mass was 1.06 (SE ± 0.02) mg seed^−1^, with a size of 1.04 (SE ± 0.01) mm wide by 2.25 (SE ± 0.02) mm long (Figure 1). Initial laboratory testing of 500 *P. lanceolata* seeds indicated 66% germination.

### 2.2. Germination Evaluation

*Plantago lanceolata* germination data for this analysis contained non-zero (i.e., ≥1) numbers; only the newly germinated *P. lanceolata* seed since the previous evaluation in each replication was used for statistical analysis. ANOVA data of the germination rate across experiments indicated no differences; therefore, data for germination were combined across the experiments. For Equation (1), the Lorentzian regression models and estimates for *a, b* and *c* parameters had *p* ≤ 0.0001, indicating that they contributed significantly to the model [16].

**Figure 2:**
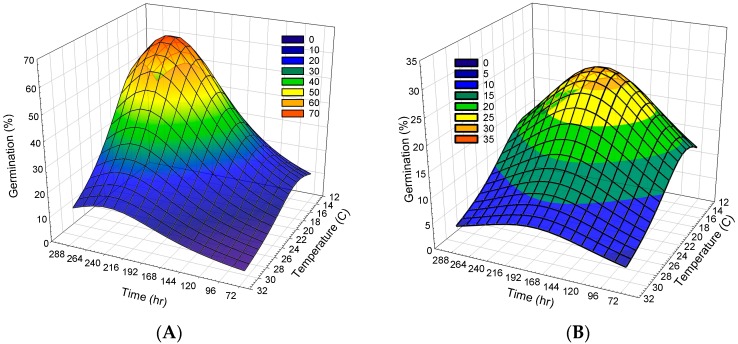
Temperature and time for cumulative (**A**) and maximum (**B**) seed germination using Lorentzian regression for *Plantago lanceolata* L. using a thermal gradient assay for seed from a *Carya illinoinensis* grove population in Webster County Georgia. Legend colors, blue hue represents lower germination with green to yellow to red hues indicating increased germination: (**A**) Cumulative germination (%) $z=\frac{65.8}{\left[\left(1+{\left(\left(x-17.8\right)/7.7\right)}^{2}\right)\;*\;\left(1+{\left(\left(y-242\right)/93.6\right)}^{2}\right)\right]}$ (*n* = 1299); (**B**) Maximum germination (%) $z=\frac{27.5}{\left[\left(1+{\left(\left(x-17.0\right)/8.5\right)}^{2}\right)\;*\;\left(1+{\left(\left(y-187\right)/118\right)}^{2}\right)\right]}$ (*n* = 1299).

For the Lorentzian regression model, cumulative *P. lanceolata* seed germination was 66% occurring at 17.8 °C at 242 h (Figure 2A). With respect to individual *P. lanceolata* seed germination counts at specific times over the 288 h time period across the experiments, the Lorentzian model maximum was 27% occurring at 17.0 °C, 187 h after initiation (Figure 2B). The 70 year average maximum and minimum temperatures for the Newman weather station at Dawson Georgia 25 km away, closest to where these seeds were collected in Webster County, for Aug, Sept, Oct and Nov were 33.0, 30.7, 26.1, and 20.9 C (maximum), and 21.0, 18.5, 12.5, and 7.4 C (minimum), respectively [17]. 

### 2.3. Greenhouse and Field Experiments

For the greenhouse experiment, indaziflam alone post-emergent at 37 g ha^−1^ significantly reduced the plant dry (DW) weight of *P. lanceolata* to 10 mg plant^−1^, providing 81% control (Table 1), as compared to the nontreated control. The addition of 2,4-D to indaziflam reduced the DW to 0 mg plant^−1^ with 99% control. For the field experiment, indaziflam alone or with 2,4-D provided season-long control of 82 to 90% at 205 d after treatment (DAT), respectively (Table 1). These data indicate that indaziflam could be used to control *P. lanceolata* even if it has already emerged, but also provide residual activity. Post-emergent applied indaziflam was more effective on seedling vs. rosette plant growth stages, and the addition of 2,4-D improved control, but not significantly.

In the greenhouse experiment, halosulfuron post-emergent applied at 26 g ha^−1^ to *P. lanceolata* DW biomass was 1456 mg plant^−1^ and did not differ from the nontreated control at 1647 mg plant^−1^ (Table 1). The addition of 2,4-D did reduce the DW biomass, but not significantly, and the control was similar at 56 and 59% for halosulfuron treatments. Similar results were seen in the field experiments where halosulfuron reduced *P. lanceolata* by 48 and 56% without and with 2,4-D, respectively, at 205 DAT.

In the greenhouse study, simazine at 1120 g ha^−1^ reduced the biomass of *P. lanceolata* significantly, applied alone or in combination with 2,4-D, as compared to the nontreated check (Table 1). Control ratings for simazine without and with 2,4-D of 78 to 83%, respectively, were not different from indaziflam treatments. Although control ratings were similar, biomass for *P. lanceolata* receiving simazine alone had greater overall biomass at 653 g plant^-1^ when compared to the indaziflam treatment of 10 g plant^−1^. For simazine treatments in the grove experiment, the addition of 2,4-D did not improve the control of *P. lanceolata* of 62 and 67%, respectively.

*Trifolium repens* L., cv. ‘Durana’ [18] stand varied by experiment each year and stand reductions were not observed for any herbicide treatment as compared to the nontreated control (data not shown). White clover exhibited tolerance to post-emergent applications of simazine and halosulfuron-methyl [19], but further research with indaziflam is needed.

## 3. Discussion

### 3.1. Seed Morphology and Germination

Even though these data are limited to one season and one population, they are similar to other research on *P. lanceolata* using the same collection methods [11,13,20,21]. Seed mass, size, and germination response were similar to that which has been previously reported for *P. lanceolata* [20]. Lacey [20] reported similar *P. lanceolata* seed sizes of 0.8 to 3.1 mg seed^−1^ with variation related to phenotype, with seed germination of 50%. Pons and van der Toon [11] noted 46 to 64% *P. lanceolata* seed germination.

The predictive results for the Lorentzian regression model cumulative *P. lanceolata* seed germination was 66%, occurring at 17.8 °C at 242 h (Figure 2A). This indicates that *P. lanceolata* germination and plant establishment can vary depending on the environment to which the seeds are exposed. In southeast USA *C. illinoinensis* groves, shorter days begin to occur after the summer solstice in June, and, along with shading from the trees, can reduce soil temperatures [22], which could initiate *P. lanceolata* seed germination. Based on the findings from these research studies and the research of others, these late summer/autumn diurnal temperature fluctuations would promote *P. lanceolata* seed germination [23]. In contrast, Thompson and Grime [12] noted that *P. lanceolata* seeds were insensitive to temperature fluctuations. While differences have been observed, the timing of herbicide applications in *C. illinoinensis* groves should align with this time in order to control *P. lanceolata* in the *Trifolium* species areas. The results of this study suggest that herbicide applications should be made during the time when diurnal temperatures are between 15 and 30 °C while abiding pre-harvest interval restrictions. Applications of herbicides with post- and pre-emergent activity may aid in controlling emerged weeds and reduce further weed emergence during the autumn of that year.

### 3.2. Herbicide Response

These data indicate that for optimum *P. lanceolata* control in *C. illinoinensis*, an October application with indaziflam would be most beneficial and the addition of 2,4-D is needed to enhance control to 90%, and as has been previously reported [13]. Halosulfuron did not provide adequate control, as reported in regards to other sulfonylurea herbicides exhibiting varying levels of *P. lanceolata* control [24]. This study further confirms that halosulfuron provides poor control of *P. lanceolata* and should not be considered for control in *C. illinoinensis* even with the addition of 2,4-D. As previously described, McCullough et al. [13] treated field-transplanted rosette stage *P. lanceolata*, which resulted in 36% control with simazine at 1120 g ha^−1^. Greater control in the *C. illinoinensis* grove was observed and may be contributed to *P. lanceolata* plants that were never stressed, as they were with transplanting for the McCullough et al. [13] study. Greenhouse control was greater than the grove experiment with the addition of 2,4-D, primarily due to environmental conditions and age of the plants. All greenhouse plants were in the 4 to 8 leaf growth stage and were well maintained, while the plants in the grove experiment were similar in size but had been exposed to greater environmental changes that harden plants and makes them more difficult to control.

The addition of 2,4-D did not improve the overall performance of any pre-emergence herbicide for *P. lanceolate* control (Table 1). Established white clovers exhibit injury from 2,4-D at 560 g ha^−1^, but recover in forage settings [25]. For the current research, 280 g ha^−1^ of 2,4-D was applied as it has activity with indaziflam in turf [13] and is registered for use in *C. illinoinensis*. Research on increasing the 2,4-D rate to 560 g ha^−1^ should also be considered as *Trifolium repens* tolerance should be further evaluated.

There are many unknown effects of *P. lanceolata* in *C. illinoinensis* production. *Plantago lanceolata* could serve as a host for insects, diseases, or nematodes that could negatively impact production. Future research should focus on evaluating herbicides registered for use in *C. illinoinensis* as a means to control weeds in the established understory, without detriment to N-producing *Trifolium* species.

### 3.3. Conclusions

*Plantago lanceolata* is an encroaching species that establishes as a winter weed. Seed testing at 13.5 to 30.5 °C indicated germination varied across temperatures with a maximum of 27% at 17.0 °C. To improve *P. Lanceolata* control in *C. illinoinensis* production, the timing of herbicide applications should be planned in autumn when diurnal temperatures are between 15 and 30 °C. Herbicides with pre- and post-emergent application timing activity may aid in controlling *P. lanceolata*, and reduce further weed emergence during the autumn of that year. 

## 4. Materials and Methods

### 4.1. Seed Collection

*Plantago lanceolata* seeds were collected from a *C. illinoinensis* grove in Webster County Georgia (31.984° N, −84.616° W) on 16 June 2015 with a Hege 125B small plot combine (Hege Company, Waldenburg, Germany) with a concave cylinder. Plants were harvested based on a visual assessment of plant maturity. Plants were considered mature and were harvested when seed heads were greater than 50% desiccated. Seed stalks were mechanically harvested with the combine, collected into nylon bags, and then dried at ambient temperature by placing in a forced air chamber with no supplemental heat. After drying, seeds were mechanically cleaned over a series round hole and slotted sieves (Clipper Seed Cleaner, A.T. Ferrell Company, Bluffton, IN, USA), and the remaining chaff removed with forced air (Oregon Seed Blower, Hoffman Manufacturing, Corvallis, OR, USA). Cleaned seeds (Figure 1) were placed in cold storage at 5 °C in a controlled environment, where they remained until testing.

Seed mass was determined by randomly counting 100 seeds, 10 different times, and weighing them as lots to obtain the average. Seed length and width were determined by physical measurement with a mm caliper using 10 seeds, 10 different times, to obtain the averages.

### 4.2. Temperature and Germination Evaluation

A thermal gradient table was constructed from a solid aluminum block measuring 2.4 m long by 0.9 m wide by 7.6 cm thick with a mass of 470 kg. On each end of the aluminum blocks, a 1.0 cm hole was drilled across the side section to allow fluid to be pumped into the table. One side contains a chiller set at 12 °C and on the other side, a heating unit at 32 °C. Each unit pumped a mixture of ethylene glycol plus water (1:10 mixture) at a rate of 3.8 L per min to generate the thermal gradient. The solutions from the chiller and heating units were independent of each other and never mixed. A grid pattern consisting of 10 cm by 10 cm cells allowed for even distribution of individual Petri dishes. This resulted in 24 increments across the established temperature gradient, with nine cells at each temperature [26,27], resulting in 216 total cells. Thermocouples made from duplex insulated PR-T-24 wire (Omega Engineering, Inc., Stamford, CT, USA) were mounted to the underside of the table in the center of each cell. The wire was inserted vertically into a hole on the underside of the table held in place by a washer and screw. Holes measured 8 mm wide by 7 cm deep to allow the thermocouple to be within 5 mm of the upper table surface at 10 cm intervals along the length of the table. The temperature was monitored continuously for each thermocouple and recorded at 30 min intervals with a Graphtec data logger (Graphtec America, Inc., Irvine, CA, USA). The data indicated a continuous temperature gradient ranging from 13.5 to 30.5 °C, resulting in changes of 0.7 °C in each cell along the length of the table.

To determine the optimal germination temperature of *P. lanceolata*, 20 seeds were evenly distributed on germination paper (SDB 86 mm, Anchor Paper Co., St. Paul, MN, USA), which was placed in a 100 by 15 mm sterile plastic Petri dish (Fisher Scientific Education, Hanover Park, IL, USA). To initiate germination, 10 ml of distilled water was added to each Petri dish and covered with the Petri dish lid to prevent evaporation. A single Petri dish was then placed in a designated cell at each 0.7 °C increment every 10 cm along the length of the thermal gradient table described above. Three dishes were placed at each temperature for a total of three replications and 72 dishes per experiment. A row across the table ranging from 13.5 to 30.5 °C with 24 Petri dishes was considered one replication (*n* = 480 seeds). The experiment was conducted two times, resulting in *n* = 2880 seeds tested. Germination counts were taken at 24, 48, 72, 96, 120, 168, 192, and 288 h. *P. lanceolata* seed germination was counted at each data collection timing. A seed was counted as viably germinated when the radicle extended more than 10 mm beyond the seed. Once counted as germinated, the seed was removed from the Petri dish. Previous research suggested *P. lanceolata* seeds with radicles longer than 1 mm from the seed coat were considered germinated [28]. However, the authors chose to wait until radicles reached 10 mm to ensure the seeds were germinated with the potential to produce a seedling and suicidal germination did not occur [23]. All counts were conducted in less than 1 h each day at approximately the same time, and the collection timing was based on when the experiment was initiated on day zero. Counts were conducted from the 13.5 °C end of the table working toward the 30.5 °C end of the table. Germination data were converted to a percentage and cumulative germination was determined for each Petri dish over the duration of that assay. Temperature data were recorded by the data loggers for each experiment. Data included temperature maximum and minimum (±0.5 °C for each thermocouple) by individual Petri dish. The highest and lowest temperature readings recorded during one germination experiment for an individual Petri dish were considered the maximum and minimum temperatures, respectively. As seed germination can be evaluated across a wide range of temperatures on the thermal gradient table simultaneously, data generated can be used to relate to the measured temperatures from the environmental conditions at various weather stations.

### 4.3. Greenhouse Experiments

Initial laboratory testing of 500 *P. lanceolata* seeds indicated 66% germination. After germination testing, *P. lanceolata* seed was sown into 15 cm pots filled with steam-sterilized Tifton loamy sand (fine-loamy, kaolinitic, thermic, plinthic, Kandiudult) with a pH of 6.5 and 0.8% organic matter. The pots were watered daily and fertilized with a 1% solution of 24-8-16 (Miracle-Gro, Marysville, OH, USA) weekly. After two weeks, the plants had 1–2 true leaves. The pots were then thinned by hand to five plants per pot, which constituted an experimental unit. Greenhouse temperatures were regulated with diurnal settings of 32/25 (±3.0 °C) and included supplemental light provided by metal halide growth lights for 12 h per day.

**Figure 3:**
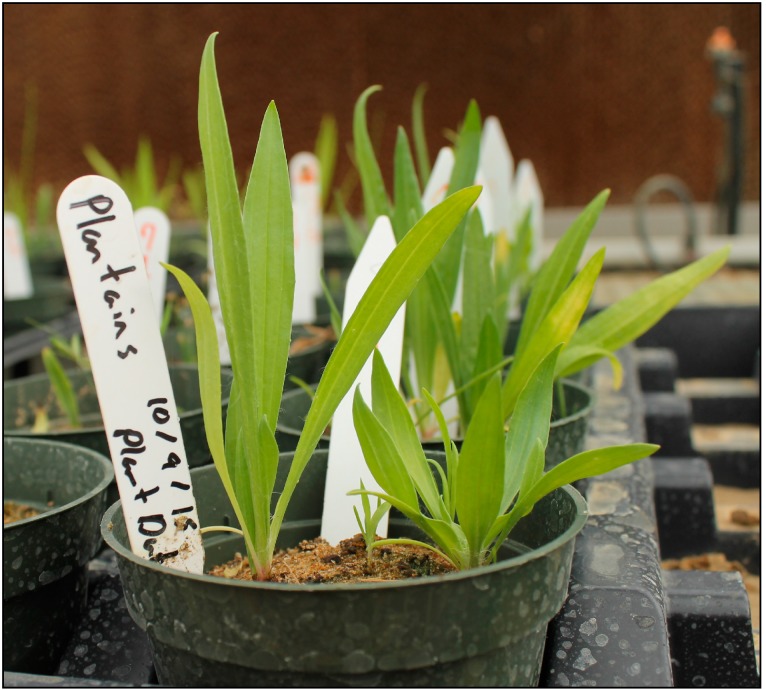
*Plantago lanceolata* L. plants tested in greenhouse experiments for response to herbicides (photograph by Sidney Cromer, used with permission).

*Plantago lanceolata* in the 4th- to 8th-leaf growth stage (Figure 3) was treated with post-emergence applications of indaziflam at 37 g ai ha^−1^, halosulfuron at 26 g ai ha^−1^, and simazine at 1120 g ai ha^−1^ alone, or in tank-mixed combinations with 2,4-D at 280 g ae ha^−1^. A nontreated control was included for comparison (Table 1). Foliar treatments were applied in a spray chamber, using deionized water and a compressed air sprayer calibrated to deliver 140 L ha^−1^ at 165 kPa. All treatments included a nonionic surfactant (NIS) (80-20 Chem Nut Inc., Leesburg, GA, USA) at 0.25% (v/v). After treatment, all plants were returned to the greenhouse and maintained under conditions previously described. Experiments were designed as a randomized complete block consisting of five replications per treatment and repeated thrice. Visual estimates of *P. lanceolata* control were recorded 21 DAT, using a scale of 0 (no control) to 100% (plant death) [29]. At 21 DAT, *P. lanceolata* plants were excavated from the pots by washing all soil free from the roots. Then the total biomass was harvested, dried in a forced air drier at 50 °C for 72 h and then weighed.

### 4.4. Field Experiments

Herbicide experiments were conducted in two areas of the same 15-year-old *C. illinoinensis* grove in 2015 to 2016 and 2016 to 2017 near Weston in Webster County Georgia at 31.984° N, −84.616° W. The soil type was Faceville loamy sand (Fine, kaolinitic, thermic Typic Kandiudults) with 81% sand, 10% silt, 9% clay and 1.5% organic matter, with a pH of 6.1. The natural population of *P. lanceolata* infestation ranged from 5 to 20 plant m^−2^ and was intermixed with cultivated *Trifolium repens* cv. Durana [20] in the interrow. Herbicide applications were made on 13 October 2015 and 14 October 2016 to *P. lanceolata* in the 2nd- to 8th-leaf growth stage. The experimental design was a randomized complete block with four replications. Treatments were the same as previously described for the greenhouse experiment and are summarized in Table 1. Individual plots were 4 m wide by 15.3 m long. Herbicides were applied using compressed air, calibrated to deliver 140 L ha^−1^ at 270 Pka with an eight-nozzle 3.6 m long tractor-mounted boom. Visual estimates of *P. lanceolata* control were recorded at 30 and 205 DAT, using a scale of 0 (no control) to 100% (plant death) [29]. Visual estimates of Durana clover ground cover were recorded 205 DAT, using a scale of 0 (no cover) to 100% (complete cover). The final rating for both experiments was at 205 DAT.

### 4.5. Statistical Analysis

#### 4.5.1. Germination Evaluation

For all measurements, analysis of variance (ANOVA) was applied to the data combined across experiment replications in time to test for interactions. Experiments were regarded as random factors, while time and temperature were considered fixed effects. For the average germination, data were averaged over the temperature gradient before doing the ANOVA to avoid inflating the error degrees of freedom.

Temperature data were used to establish a minimum, maximum, and average temperature for each cell. The average temperature was the arithmetic mean of all temperatures for a cell over the duration of each test. Minimum and maximum temperature was the lowest and highest temperature received for that cell during the duration of a test.

#### 4.5.2. Cumulative and Maximum *P. lanceolata* Seed Germination

In order to establish the relationship between time, temperature, and buckhorn seed germination, a three-dimensional non-linear regression was conducted in SigmaPlot (SigmaPlot 14.0. SPSS Inc. 233 S. Wacker Dr., 11th Floor, Chicago, IL, USA). The non-linear relationship between the two independent variables of time and temperature were regressed to *P. lanceolata* germination. A Lorentzian distribution model,

(1) $$z=\frac{a}{\left[\left(1+{\left(\left(x-x0\right)/b\right)}^{2}\right)\;*\;\left(1+{\left(\left(y-y0\right)/c\right)}^{2}\right)\right]}$$

was fitted to the germination data. Temperature (*x*) and time (*y*) data were used to model the parameters (*a*, *b*, and *c*) to predict the maximum temperature (*x*0) and maximum time (*y*0) that produced the maximum germination (*z*), similar to other weed research [30]. Data for percent total cumulative, and by each count to establish the maximum, were modeled. The Lorentzian distribution belongs to the category of *t*-distribution with wider and more comprehensive analysis than other models when there are outlier data points [31].

#### 4.5.3. Greenhouse and Field Experiment

Field and greenhouse *P. lanceolata* control ratings, weed DW, and Durana clover ground cover were applied to the data combined across experiments, location, and year to test for interactions using an analysis of variance (ANOVA) with PROC Mixed in SAS 9.4 (SAS Institute Inc, Cary, NC, USA). Treatments were separated with a Tukey–Kramer least squares means test (*p* ≤ 0.05). Herbicide treatment was considered a fixed effect, and experiments (nested within year) and replications were regarded as random factors.

## Figures and Tables

**Table 1:** Effect of herbicide treatment on *Plantago lanceolata* L. control in greenhouse experiments and from autumn applications in *Carya illinoinensis* (Wangenh.) K. Koch groves in Webster County Georgia.

Treatment ^a^	Rate	2,4-D Amine	Greenhouse Experiments ^b,d^		Grove Experiments ^c,d^
	g ai ha^−1^		% control	dry weight mg plant^−1^	% control
Nontreated	-	-	0 c	1647 a	0 c
Indaziflam	37	0	81 a	10 e	82 ab ^c^
Indaziflam	37	280	99 a	0 e	90 a
Halosulfuron	26	0	56 b	1456 ab	48 b
Halosulfuron	26	280	59 b	1063 bc	56 b
Simazine	1120	0	78 a	653 cd	62 b
Simazine	1120	280	83 a	240 de	67 b

^a^ All applications included non-ionic surfactant at 0.25% v/v; ^b^ Greenhouse experiments were conducted three times, once in 2015 and twice in 2016, data from control ratings and biomass taken 21 d after treatment; ^c^ Oct applications of herbicides in 2015 and 2016 in two separate experiments. Data for control ratings taken 205 d after treatment combined over experiments; ^d^ Means followed by the same letters are not significantly different at *p* < 0.05 by Tukey–Kramer least square means test.
